# 842 Management of Burn and Soft Tissue Injuries During the Israel-Hamas War

**DOI:** 10.1093/jbcr/iraf019.373

**Published:** 2025-04-01

**Authors:** Shiran Katabi, Stav Sarna Cahan, Tomer Tzur

**Affiliations:** Hadassah Medical Center; Hadassah Medical Center; Hadassah Medical Center

## Abstract

**Introduction:**

The Israel-Hamas War, which started in October 2023, led to significant casualties and numerous war-related injuries. This study outlines the role of plastic surgeons during the conflict, detailing the types of burn and soft tissue injuries treated and the procedures performed.

**Methods:**

We included patients injured during the Israel-Hamas conflict who were transferred to Hadassah University Medical Center, a tertiary referral hospital, for multidisciplinary care between October 2023 and June 2024. Data was collected on epidemiology, mechanisms of injury, anatomical injury locations, types of plastic surgeries performed, the number of interventions, and any complications.

**Results:**

From the onset of the Israel-Hamas war in October 2023 until June 2024, 272 individuals with conflict-related injuries were admitted to Hadassah University Medical Center. Of these, 268 were hospitalized for observation or treatment, with 30% either admitted to the Department of Plastic and Reconstructive Surgery or to other departments requiring plastic surgeons for either conservative or surgical interventions. The injury profile revealed that over 90% of the injuries resulted from gunfire, improvised explosive devices, rocket shrapnel, and burns. Procedures included standard interventions such as debridement, skin grafts, and flap reconstruction, as well as more advanced techniques like laser therapy and hyperbaric treatment.

**Conclusions:**

This study highlights the injury profile of the conflict from the perspective of plastic and reconstructive surgeons. It provides a comprehensive overview of representative cases and examines the range of treatment approaches available in a modern plastic surgery department at a tertiary referral hospital.

**Applicability of Research to Practice:**

This study provides practical insights into managing war-related burn and soft tissue injuries, emphasizing advanced plastic surgery techniques like laser therapy and hyperbaric treatment. It highlights the importance of multidisciplinary care and early intervention, offering guidance for trauma surgeons in both military and civilian settings.

**Funding for the Study:**

N/A

